# Evaluation of Radio Communication Links of 4G Systems

**DOI:** 10.3390/s22103923

**Published:** 2022-05-22

**Authors:** Mouloud Ayad, Reem Alkanhel, Kamel Saoudi, Mourad Benziane, Smail Medjedoub, Sherif S. M. Ghoneim

**Affiliations:** 1 Department of Electrical Engineering, Faculty of Sciences and Applied Sciences, University of Bouira, Bouira 10000, Algeria; k.saoudi@univ-bouira.dz (K.S.); s.medjedoub@univ-bouira.dz (S.M.); 2 Department of Information Technology, College of Computer and Information Sciences, Princess Nourah bint Abdulrahman University, P.O. Box 84428, Riyadh 11671, Saudi Arabia; 3 Electrical Engineering Department, College of Engineering, Taif University, P.O. Box 11099, Taif 21944, Saudi Arabia; s.ghoneim@tu.edu.sa

**Keywords:** radio communication systems, 4G systems, data analysis

## Abstract

Radio communication systems are very widely present in our current smart lifestyles. It consists of two ends, which can carry the transmitter’s information to the receptor. Before installing any radio communication system, it is necessary to analyze the link resources. Hence, this analysis allows the determination of the received radio communication strength to prove if it is sufficient for the link to work correctly and assure a high quality of service. For this reason, new services and technologies are integrated. The objective of the present work is to improve the performance of the radio communication link of 4G systems. The study is based on real measurements using the drive test. The data collected by the drive test are analyzed to increase the performance of the radio communication. Based on this data analysis, recommendations and suggestions are issued for improving the radio communication link. The obtained results indicate a significant amelioration in the performance of the radio communication link.

## 1. Introduction

The field of telecommunications has experienced technological progress in recent years thanks to the strong demand from the population and industry. Among the concerns in this area are mobile networks. The development of mobile networks has continued to grow, and several generations have appeared (1G, 2G, 3G, 4G, …). For example, the 4G Long Term Evolution (LTE) network allows a very high-speed mobile. The evolution of these generations leads to the development of mobile network architecture and the integration of new services. This development affects the mobile network infrastructures such as switching centers, radio communication systems, etc. A radio communication system comprises two ends, allowing information to be transported from one end to another. Before installing a radio communication system, the calculation of the radio communication budget is necessary. Indeed, this calculation makes it possible to determine whether the level of power received by the receiver will be sufficient for the link to operate correctly.

In the literature, several studies have been presented based on the existing propagation path loss models in diverse propagation environments (suburban, dense urban, rural). The authors in the paper [1], present a prediction model to predict the path loss. In order to validate the proposed model, the authors give a comparative study to the models of Okumura and COST–Hata. Based on the Okumura–Hata model and free space path loss, Vithanawasam et al., in [2], are calculated the data rates for varying scenarios in different environments (urban, suburban, and rural). The authors in [3] present five used radio propagation models: Okumura–Hata model, Hata short-range devices model, Cost231Hata model, extended Stanford University interim model, and standard propagation model. Okumura–Hata, Ericsson 9999, Lee propagation, and ECC-33 models are used in [4] for the urban traffic noise monitoring system. The path loss models used to evaluate the study in the paper [5] are Okumura–Hata models, COST231 Hata models, IEEE 802.11ah models, and COST231 Walfisch–Ikegami model. In [6], the path losses calculation is based on the Okumura–Hata model.

In [7], to obtain the relationship between the distance and the measurement, the authors used multivariable fingerprints to study the path-loss model.

Other models, close-in, floating-intercept, and alpha-beta-gamma models, are used in [8]. These models are used also in [9,10,11]. The procedures of these models are presented in these two papers [8,9,10]. The path loss model in [12] is based on the received signal strength indicator measurements. It is a Bayesian particle filter used for overcomes the noise caused by obstacles and sudden signal fading. The power of pure chirp signals from a long-range end-devices radio is applied in [13] to avoid missing information and inaccurate path loss exponent values. It is a case study of point-to-point Internet of Things radio networks measurement.

For the mobile operators, they use most of these propagation models.

This work aims to study and evaluate the radio communication links of 4G LTE systems. Based on the analysis of measurement data, the performances of the radio communication are ameliorated by planning and dimensional mobile network on the base of real measurements of the different data. For this purpose, this work is divided into three parts as follows: firstly, the theoretical background on the basic concepts of radio communication links is introduced. The second part introduces the methodology and material used. The results and discussion are presented in the third part. Finally, the paper is concluded by the conclusion.

## 2. Background

A radio communication link transports information from a transmitter to a receiver. The installation of such a link requires a calculation to improve its performance. This chapter will present the process of calculating the radio communication links of 4G systems. Therefore, the link budget makes it possible to calculate the maximum permissible propagation losses denoted MAPL (maximum allowable power losses) for a mobile located at the edge of the cell, which can reach it while maintaining the level of sensitivity of the base station. It predicts cell coverage radius, propagation pattern, transmitter, and receiver parameters. The transmit power is the maximum power of the base station (eNB), having a typical value for the macro cell (43–46) dBm at the antenna connector. Antenna gain is the power radiated by the antenna in a given direction relative to the energy released by a perfect unidirectional antenna that radiates evenly in all directions. It is expressed in dB but to indicate that the reference antenna used is isotropic, it is customary to speak of dBi. A concept related to that of gain is the effective area of an antenna, which connects to the size and shape of the antenna. The relationship between the gain and the effective area of the antenna is given by [14]:

(1) $$G=4\pi A/\lambda^{2},$$

With:

A: Equivalent area of the antenna.

λ: Length of the radiated wave.

The gain of a typical base station antenna is between (15 to 18) dBi, and the gain of the UE (user equipment) antenna is in the range of (−5 to 10) dBi.

The link budget of the transmission system for all gains and losses is the total received signal. It is the received power Pr expressed in dBm by the following equation [15]:

Pr = Pt + Gr + Gt − L,(2)

where:

Pt: transmitter power in dBm.

Gr: receiver antenna gain in dB.

Gt: transmitter antenna gain in dB.

L: loss in the considered direction.

A receiver is characterized by its sensitivity (S) which is the minimum power level of the received signal that allows the reception of the signal (minimum reception power level) [16]. It is given by the following formula:

S = 10 log(K. T. BRx) + SINR_required_ + NF, (3)

With:

K: Constant of Boltzmann (1.388062 × 10^−23^).

T: Ambient temperature (Kelvin).

BRx: Receive bandwidth.

SINR_required_: The value of the SINR needed at the receiver is an indicator of system performance; the lower it is, the more efficient the system, depending on the number of resource blocks, information rate required, etc.

NF: RF Noise Figure (dB): depends on the duplexing mode and the duplex deviation.

4G operates in several bandwidths and the bandwidth selection relates directly to base station capacity. During practical planning, for a first-time deployment, the dimensioning is done by a bandwidth of 5 MHz and 10 MHz.

### 2.1. Model of the Propagation

The propagation model makes it possible to estimate the value of the path attenuation. There are several types of models: an empirical model is a mathematical formula used to predict the impact of a transmitter on a specific reception area, andphysical models predict the radio waves propagation and calculate the paths of radio waves taking into account reflection and diffraction phenomena. For our study, we will choose the empirical propagation model. Its formula depends on several factors, namely: the frequency of the wave; the heights of transmitter and receiver antennas, and the distance.

#### 2.1.1. Free Space Propagation

We speak of propagation in free space when there is direct visibility between receiving and transmitting antennas in the absence of any obstacle. The following formula provides the attenuation [1,2,3]:

L = 32.4 + 20log(f) + 20 log(d),(4)

With:

f: operation frequency (MHz)

d: distance between receiver and transmitter (km).

#### 2.1.2. Okumura–Hata Model

The best-known empirical model is the Okumura–Hata model, which is based on Okumura measurements taken in the Tokyo area of Japan. This model considers several factors, essentially the nature of the environment, by specifying its degree of urbanization (dense urban, suburban, rural). The Okumura–Hata model is adapted for the limits:

- Frequency range: (150 to 1000) MHz
- Height of the mobile terminal (H_m_): (1 to 10) m
- Height of the base station (H_b_): (10 to 200) m
- Distance (d): (1 to 20) km

For this model of Okumura–Hata, the equation of path loss is given by the following equations:

- For the urban environment [2,4,17,18,19]:

Lu = 69.55 + 26.16 log(F) − 13.82 log(h_b_) − a(h_m_) + (44.9 − 6.55 log(h_b_)) log(d) (5)

With: a(h_m_) = (1.1 log(F) − 0.7) h_m_ − (1.56 log(F) − 0.8).

- For dense urban environment:

Lud = Lu = 69.55 + 26.16 log(F) − 13.82 log(h_b_) − a(h_m_) + (44.9 − 6.55 log(h_b_)) log(d) (6)

With a(h_m_) = 8.29 log(1.54 h_m_)^2^ − 1.1.

Suburban environment:

Lsu = Lu − 2(log(F/28))^2^ − 5.4.

- For the rural environment:

Lr = Lu − 4.78 (log(F))^2^ + 18.33 log(F) − 35.94 (7)

With:

F: Designates the frequency (MHz).

h_m_: Designates the size of the mobile terminal (m).

h_b_: Designates the height of the base station (m).

d: Designates the distance (km).

a(h_m_): Correction factor for the height of receiver antenna.

#### 2.1.3. Cost 231Hata Model

The model of Cost 231 Hata is a widely used model. This model is introduced by M. Hata in 1980 [19]. This model has the following parameters:

- Transmitter height (30 to 100) m.
- Frequency range (500 to 2000) MHz.
- Size of UE (1 to 10) m.
- Link distance (>20 km).

The path loss equation of this model is given in the following equation [1,5]:

Lu = 46.3 + 33.9 log(F) − 13.82 log(h_b_) − a(h_m_) + (44.9 − 6.55 log(h_b_))log(d) + C (8)

a(h_m_) = 1.1 log(F) − 0.7 × h_m_ − (1.56 log(F) − 0.8); for suburban and rural environment

a(h_m_) = 3.2(log(11.75 × h_m_))^2^ − 4.97; for urban environment

where C is the correction factor set to 3 dB for urban locations and 0 dB for rural or suburban locations.

F: Designates the frequency (MHz).

h_b_: Refers to the height of the base station (m).

d: Refers to the distance (km).

h_m_: Designates the height of the mobile terminal (m).

a(h_m_): Correction factor for the height of receiver antenna.

### 2.2. Study and Simulation of the Radio Links of a 4G System

The radio link quality of 4G LTE systems depends on a set of parameters such as propagation model, variation of the distance between transmitter and receiver, the height of the eNB, power supply, and others. This part presents the simulation results obtained by varying these different parameters and their influences on the radio communication links. In this study, we are interested in a Maximum Path Loss (MAPL). From the MAPL can calculate the radius of the cell to be discovered, we have already given an example of the calculation of coverage radius in the downlink interface. In this example, we have chosen the propagation model Cost 231 Hata, which estimates the total number of sites to avoid a communication cut.

#### 2.2.1. Cost 231Hata and Okumura–Hata Models

**Figure 1:**
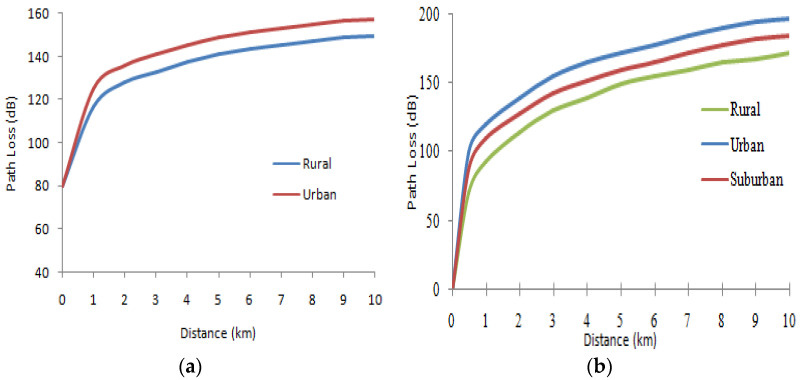
Influence of distance and type of medium on propagation: (**a**) Cost 231 Hata model; (**b**) Okumura–Hata model.

The model of Cost 231 Hata is used to calculate path loss in a UE system. The influence of different parameters on the Lx signal attenuation with the Cost231Hata and Okumura–Hata model is given. We have given the variation of the distance between receiver and transmitter. The variation range of the distance separating the receiver and the transmitter is: [0.01 km, 10 km]. The other parameters are set as follows: h_b_ = 80 m; h_m_ = 2 m and F = 1000 MHz. The obtained results are presented in Figure 1:

We note a proportional relationship between the separation distance and the attenuation (that the attenuation increases with the increase in the distance separating receiver and transmitter in the two media).

**Figure 2:**
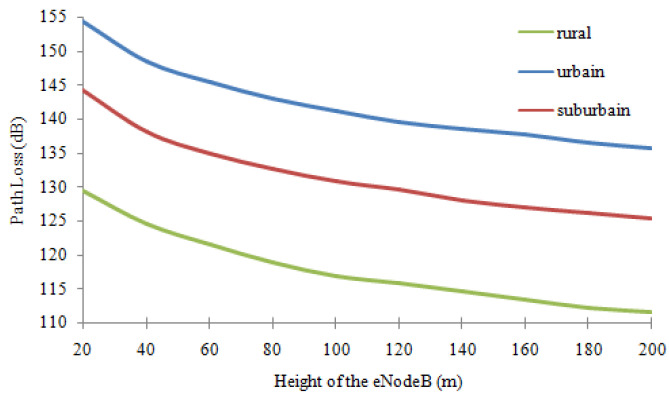
Influence of distance and type of medium on propagation.

The influence of the height of the eNB is presented in Figure 2. In this case, the range of variation of the height of the eNB is [20 m, 200 m]. The other parameters are set as follows: h_m_ = 2 m; d = 2 km and F: 1000 MHz.

We notice an anti-proportional relationship between the size of the eNB and the attenuation, i.e., if H_b_ increases L_x_ decreases.

#### 2.2.2. Free Space Model

At distance R, the power density p(R,θ,φ) (number of Watts/m^2^) is given by [14]:

p(R,θ,φ) = P_a_/4πR^2^ [W/m^2^] (9)

(R) is the distance between the transmitter and the receiver. From the calculation of the sphere of the isotropic antenna and the definition of the antenna gain, it can be shown that the distance between the transmitter and the receiver introduces attenuation of the propagation due to free space (Afs) at [14,20]:

Afs = 20 log(4πd/λ) [dB](10)

**Figure 3:**
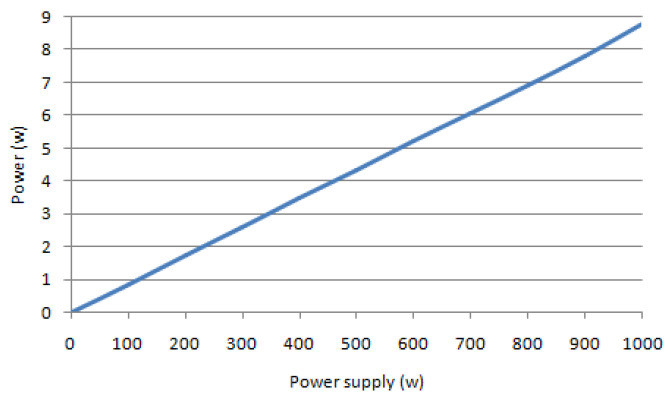
Variation of received power vs. power supply.

The variation of the power received according to the power supply from 1 Watt up to 1 kW is presented in Figure 3. The simulation parameters are as follows: P_a_ = [1, 1000] Watt; d = 3 km and λ = 0.01 m.

Note that the relationship between P and P_a_ is linearly proportional because the function is a straight line.

**Figure 4:**
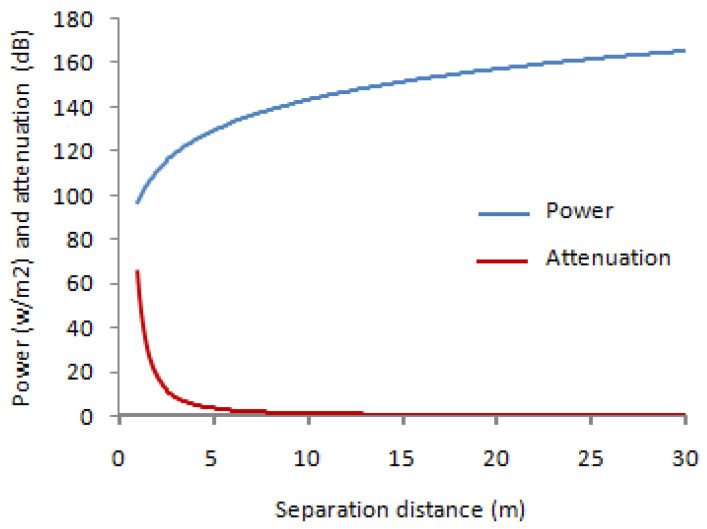
Relationship between power and attenuation vs. distance.

The variation of received power and attenuation vs. distance (with: Pa = 1000 Watt; d = [0, 30] m and λ = 0.1 m) are given in Figure 4.

We notice a proportional relationship between attenuation and distance, while the relationship between power and distance is anti-proportional.

**Figure 5:**
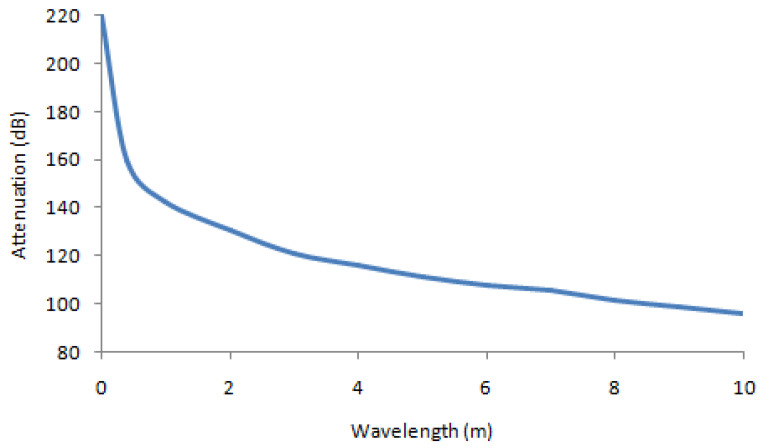
Variation of attenuation vs. wavelength (λ).

The variation of attenuation according to wavelength for the parameters: Pa = 1000 Watts; d = 100 m and λ = [0.01, 10] m is presented in Figure 5:

#### 2.2.3. Discussion of the Results

According to the obtained results, the types of the medium on which the electromagnetic waves propagate directly influence the weakening of the signal. At the same time, the propagation is better when the obstacles are reduced. In conclusion of this part, we can increasingly classify the quality of propagation as poor quality as follow:

1. Urban environment.
2. Dense urban environment.
3. Suburban environment.
4. Rural environment.

The distance directly affects the received signal quality. As a result, for 4G LTE networks, the distance between the eNB and the mobile terminal must be considered to guarantee an acceptable link quality.

For the height of the eNB and mobile terminal and according to the obtained results, it can conclude that the link is better when the height increases either at the side of the terminal or the eNB or both. This is explained by the decrease in the number of obstacles with the height until the link can be considered in free space.

## 3. Materials and Methods

The function of the drive test is the evaluation of the level of fields transmitted from a transmitter at different positions within the coverage region. Data is collected for different clusters for further analysis. The drive test produces data files containing field-level measurements and GPS coordinates collected for each point.

The equipment used for the drive test are:

- The GENEX PROBE V3.5 software (installed on a laptop and which records the data received).
- The GPS gives each signal capture point a geographical location.
- The vehicle with all facilities.

To obtain best measurements, the above conditions must be respected:

- Conduct at moderate speed (less than 50 km/h).
- Stay in the same over floor class whenever possible.
- Cross route for one time and repeat the measurements in the case of errors.

The TEMS Investigation is an excellent tool for verifying, optimizing, and maintaining wireless networks. TEMS Investigation is a complete solution for all of a carrier’s wireless network optimization tasks. With TEMS surveys, operators can achieve better service performance, better accessibility, and improved voice quality. The TEMS is used for:

- Tune and optimize networks.
- Detect faults and troubleshoot wireless networks.
- Verify actual endpoint performance with UE.
- Check coverage cell, accessibility, capacity, and integrity.

**Figure 6:**
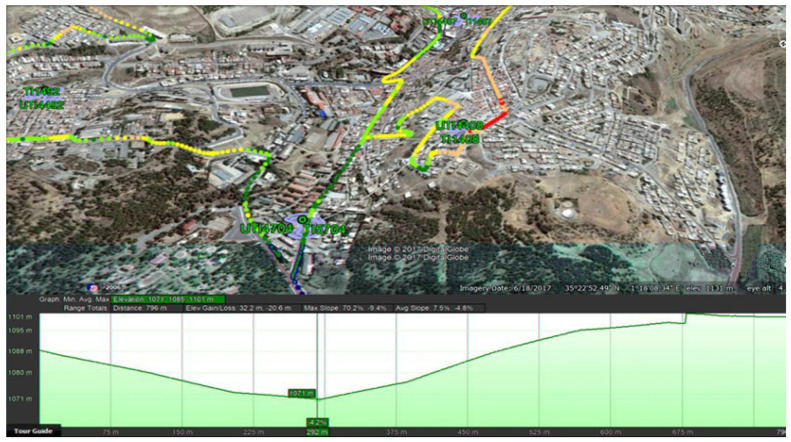
Studied area.

The location of the study area (Tiaret-Algeria) is 35°22′52″ N and 1°18′08″ E. Figure 6 presents an overview of the studied area.

Planning a 4G LTE network is a critical step. Therefore, the 4G LTE network operators must focus on planning before any implementation of their networks to avoid additional costs. The planning and dimensioning is an essential task; indeed, these fundamental tasks are a result of the optimization task. Moreover, the optimization task is based on the network’s deployment and maintenance. The first step is collecting the necessary information about the studied area. Based on the collected information, the 4G LTE network performances are tested to evaluate the quality of service (QoS) offered. If the QoS is acceptable, the studied area is approved. Otherwise, the collected information must be analyzed to improve the QoS. Based on these analyses, suggestions, and recommendations about the necessary modification and configurations that must be done are delivered. After realizing the recommended amendment, the new collection data and QoS test are required. The process is repeated until the QoS is acceptable.

## 4. Results and Discussion

In this part, we will give the different parameters used to improve the quality of service of the 4G LTE network. The used parameters in this study are: signal to interference and noise ratio (SINR), reference signal received power (RSRP), reference signal received quality (RSRQ), and debit.

### 4.1. Reference Signal Received Power (RSRP)

The RSRP is the received average power of the downlink signals. It is given by the following equation [21,22]:

RSRP = (1/K)∑P_rs,k_ (11)

where: (P_rs,k_) [Watts] is the power of the received signal.

**Figure 7:**
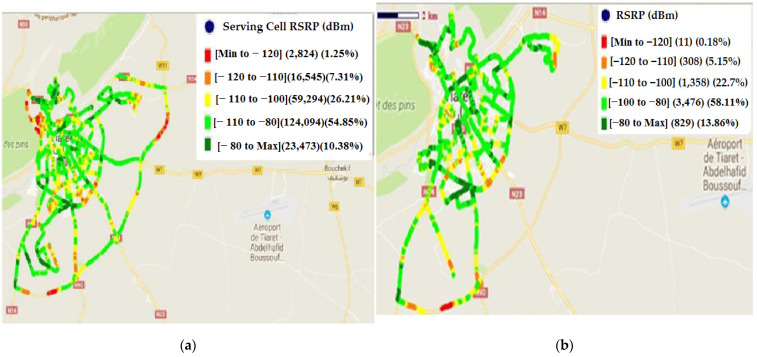
RSRP distribution: (**a**) before treatment; (**b**) after treatment.

The RSRP provides important information about the strength of the received signal to determine optimal power settings for network operation. The most studied area problems are: quality of area coverage due to the geographic profile. In addition, we have inadequate coverage in this area due to the absence of a dominant cell. The obtained results of RSRP with the drive test before dealing with the problems are presented in Figure 7.

**Figure 8:**
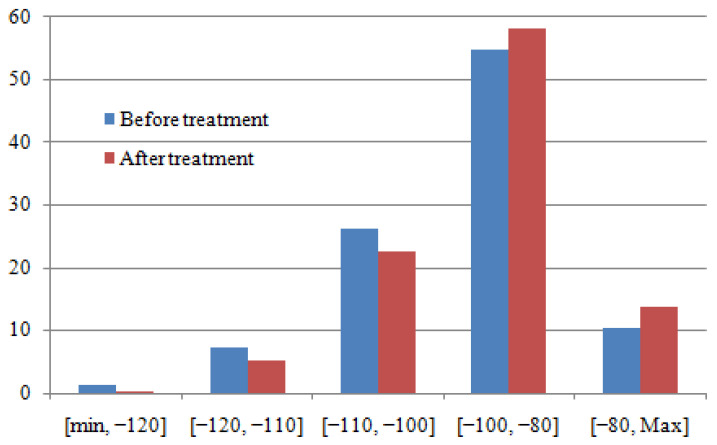
Histogram’s distribution of RSRP (before and after treatment).

Under the normal state, when the received signal is not affected by the noise and interference, RSRP varies between [−100, −80] dBm. In good condition, RSRP is higher than −80 dBm. For these two states, no action will be done. If the value of RSRP is less than −100 dBm, an activity of optimization is necessary. Several actions are possible: boost the power transmission, increase the height of the transmitter, and the implementation of new eNB if necessary. The obtained values of RSRP before and after treatment are given in Table 1:

The histogram’s distribution of RSRP before and after treatment is presented in Figure 8.

The aim of this section is the amelioration (ensuring maximum possible coverage) of the received RSRP in the area where the power is low (three first cases in Table 1). According to Figure 8 and Table 1, there is an amelioration of 6.74% in RSRP distribution. These ameliorations are distributed as follows: amelioration of 01.07% in the range of [min, −120] dBm; amelioration of 2.16% in the range of [−120, −110] dBm and amelioration of 3.51% in the range of [−110, −100] dBm.

### 4.2. Reference Signal Received Quality (RSRQ)

RSRQ is defined as the quality of the received signal. Measuring the RSRQ becomes mainly crucial near the cell’s border when decisions must be made to transfer to the adjacent cell.

The reception quality of the reference signal is used only during the connected states.

The RSRQ can be measured with the following equation [7,21,22]:

RSRQ = N_RB_ × RSRP/RSSI (12)

where: N_RB_ is the number of resource blocks of measured RSSI.

RSSI: Received Signal Strength Indication (average of the total received power).

RSRP: Reference Signal Received Power.

**Figure 9:**
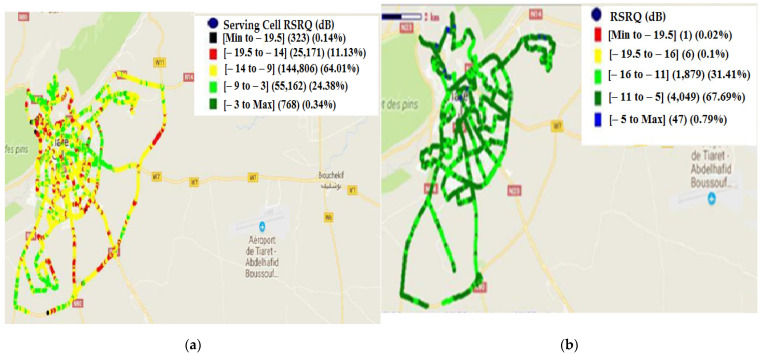
RSRQ distribution: (**a**) before treatment; (**b**) after treatment.

The obtained results of RSRQ with the drive test before dealing with the problems are presented in Figure 9.

In this case, the value of RSRQ under the normal state is [−9, −3] dB. In good condition, RSRQ is higher than −3 dB. For these two states, no action will be done.

When the value of RSRQ is less than −9 dB, an action of optimization is necessary. These actions are possible: handover management (the process of transferring intercellular of UE), increase in the height of the transmitter, optimization of antenna tilt parameters, and implementation of new eNB if necessary.

**Figure 10:**
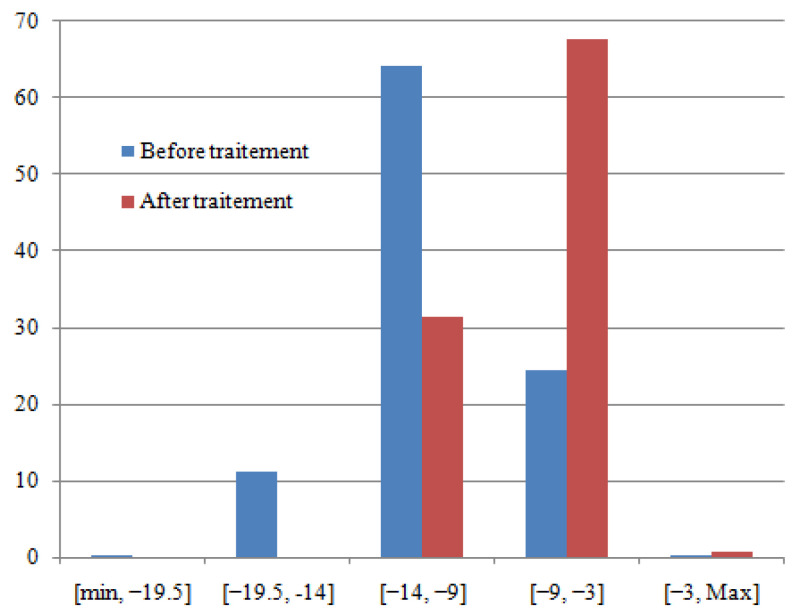
Histogram’s distribution of RSRQ (before and after treatment).

The obtained values of RSRQ before and after treatment are given in Table 2:

The histogram’s distribution of RSRP before and after treatment is presented in Figure 10.

According to Figure 10 and Table 2, the amelioration in RSRQ is 43.76%. The amelioration is distributed as follows: amelioration of 00.12% in the range of [min, −19.5] dB; amelioration of 11.03% in the range of [−19.5, −14] dB and amelioration of 32.6% in the range of [−14, −9] dB. In this case, the amelioration is very significant.

### 4.3. Signal to Interference andNoise Ratio (SINR)

SINR is a tool for measuring the quality of the UE network when the signal energy decreases with distance, i.e., the path loss of environmental parameters (for example, background noise, and the interference force of another simultaneous transmission).

The signal-to-noise ratio SINR is given by the following equation:

SINR = S/(I + N)(13)

S: Power of the received signal.

I: Interference power.

N: Noise power.

**Figure 11:**
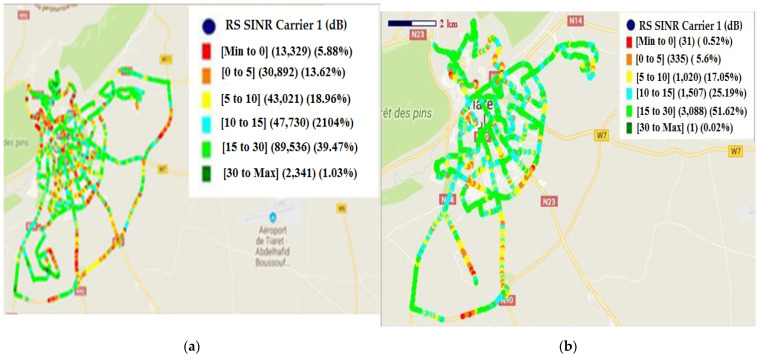
SINR distribution: (**a**) before treatment; (**b**) after treatment.

The SINR distribution before and after treatment isgiven in Figure 11.

In the case of SINR, the values in the normal state are (15, 30) dB. In good condition, the values are higher than 30 dB. When the value of SINR is less than 15 dB, an action of optimization is necessary. These actions are possible: boost the power transmission, handover management; increase the height of the transmitter, optimization of antenna tilt parameters, and the implementation of new eNB if necessary.

**Figure 12:**
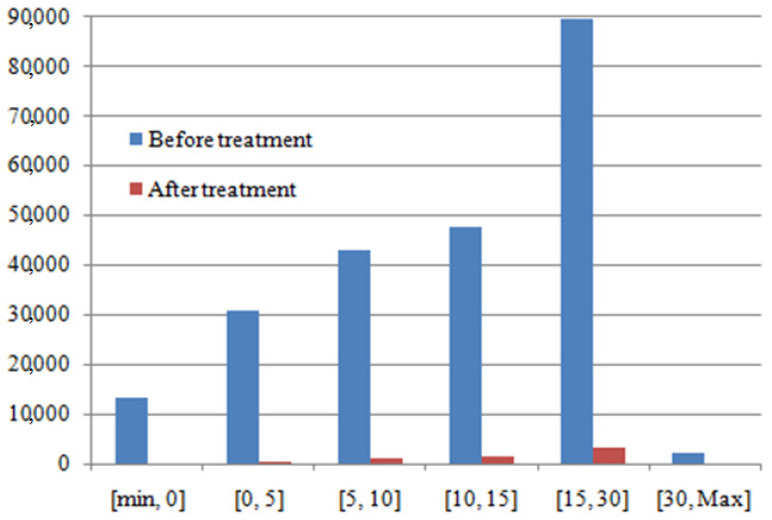
Histogram’s distribution of SINR (before and after treatment).

The obtained values of SINR before and after treatment are given in Table 3:

The histogram’s distribution of SINR before and after treatment is presented in Figure 12.

The aim is to reduce the value of the SINR. According to Figure 12 and Table 3, there is great amelioration in all ranges of the SINR. The best amelioration is in the range of [15, 30] dB. In this range, the reduction of 46,223 dB is introduced. In the ranges [<0] dB, [0, 5] dB, [5, 10] dB and [10, 15] dB, the reductions are 13,298 dB, 30,557 dB, 42,001 dB, and 46,223 dB, respectively. The weak reduction is in the range of [>30] dB (reduction of 2340 dB).

### 4.4. Debit

The debit of the user is higher than 3G (downlink of more than 300 Mbps against 14 Mbps in UMTS). The supported user rate must match the bandwidth spectrum.

For the downlink rate, the eUTRAN can use tow transmitters’ antennas for the eNB and tow receivers’ antennas for the UE.

**Figure 13:**
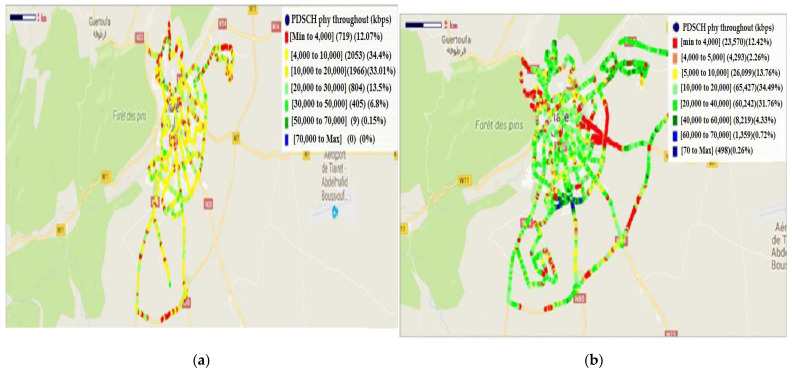
PDSCH physical throughout: (**a**) before treatment; (**b**) after treatment.

The obtained results of PDSCH (physical downlink shared channel) physical throughout before and after treatment are given in Figure 13.

**Figure 14:**
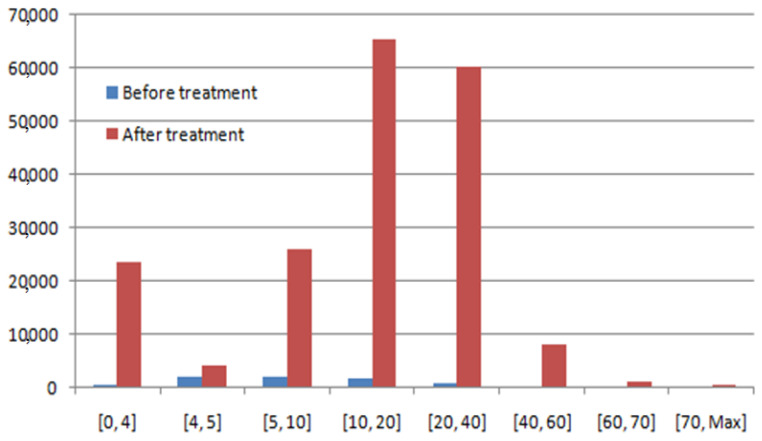
Histogram’s distribution of PDSCH (before and after treatment).

The obtained values of PDSCH physical throughout before and after treatment are given in Table 4:

The histogram’s distribution of PDSCH before and after treatment is presented in Figure 14.

The aim is the amelioration of the downlink debit. According to Figure 14 and Table 4, there is amelioration in all ranges of the downlink debit. The best amelioration is in the range of [10, 20] Mbps and the value of the amelioration is 63,461 Kbps. In the ranges of [0, 4] Kbps, [4, 5] Kbps, [5, 10] Kbps, [20, 40] Kbps, [40, 60] Kbps, and [60, 70] Kbps, the ameliorations are 22,851 Kbps, 2240 Kbps, 24,046 Kbps, 59,438 Kbps, 7814 Kbps, and 1350 Kbps, respectively. The weak amelioration is in the range of [>70] Kbps (amelioration of 498 Kbps).

**Figure 15:**
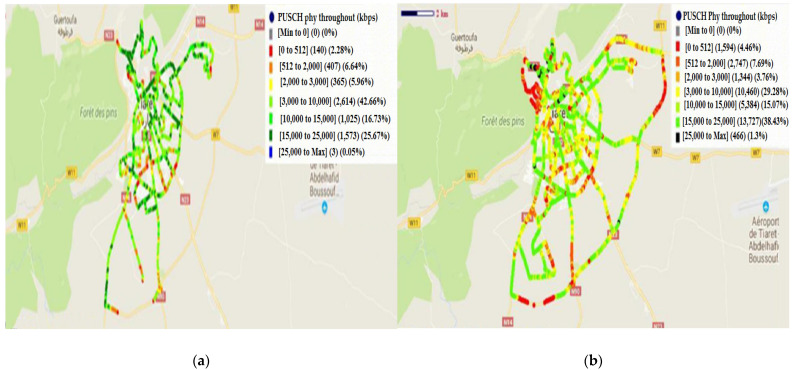
PUSCH physical throughout: (**a**) before treatment; (**b**) after treatment.

For the uplink, the obtained results of PUSCH (physical uplink shared channel) physical throughout before and after treatment are given in Figure 15.

**Figure 16:**
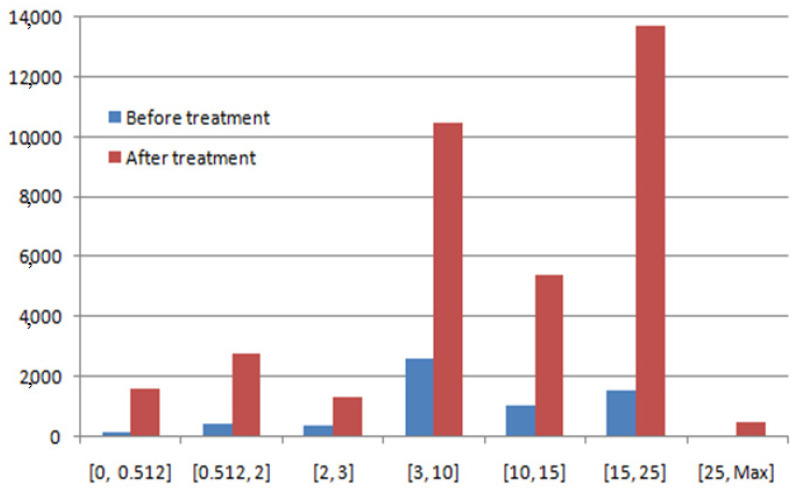
Histogram’s distribution of PUSCH (before and after treatment).

The obtained values of PUSCH physical throughout before and after treatment are given in Table 5:

The histogram’s distribution of PUSCH before and after treatment is presented in Figure 16.

Additionally, there is amelioration in all ranges of the uplink debit. According to Figure 16 and Table 5, the best amelioration is in the range of [15, 25] Mbps and the value of the amelioration is 12,154 Kbps. In the ranges of [<0.512] Mbps, [0.512, 2] Mbps, [2, 3] Mbps, [3, 10] Mbps, and [10, 15] Mbps, the ameliorations are 1454 Kbps, 2340 Kbps, 979 Kbps, 7846 Kbps, and 4359 Kbps, respectively. The weak amelioration is in the range of [3, 10] Kbps (amelioration of 463 Kbps).

## 5. Conclusions

Mobile telecommunications networks have experienced significant development. The 4G LTE is one of these networks. The effect of this network leads to the integration of new services to ensure high speeds. This development also affects the various network infrastructures. The planning and optimization phase provides this development. Based on drive test tools and TEMS, this work has studied the evaluation of a 4G LTE network. A set of tests and measurements of the several parameters (received signal quality, debit, etc.), have been carried out to find solutions to any problem leading to a weak network. The obtained results demonstrate amelioration in the 4G LTE network. After treatment, the used parameters are enhanced, and the quality of service becomes better than before treatment.

## Figures and Tables

**Table 1:** RSRP values before and after treatment.

Range	RSRP (dBm)	
	Before Treatment	After Treatment
[min, −120]	28,24 (1.25%)	11 (0.18%)
[−120, −110]	16,545 (7.31%)	308 (5.15%)
[−110, −100]	59,294 (26.21%)	1,358 (22.7%)
[−100, −80]	124,094 (54.85%)	3,476 (58.11%)
[−80, Max]	23,473 (10.38%)	829 (13.86%)

**Table 2:** RSRQ values before and after treatment.

Range	RSRQ (dB)	
	Before Treatment	After Treatment
[min, −19.5]	323 (00.14%)	1 (00.02%)
[−19.5, −14]	25,171 (11.13%)	6 (00.10%)
[−14, −9]	144,806 (64.01%)	1879 (31.41%)
[−9, −3]	55,162 (24.38%)	4049 (67.69%)
[−3, Max]	768 (00.34%)	47 (00.79%)

**Table 3:** SINR values before and after treatment.

Range	SINR (dB)	
	Before Treatment	After Treatment
[min, 0]	13,329 (5.88%)	31 (00.52%)
[0, 5]	30,892 (13.62%)	335 (05.60%)
[5, 10]	43,021 (18.96%)	1020 (17.05%)
[10, 15]	47,730 (21.04%)	1507 (25.19%)
[15, 30]	89,536 (39.47%)	3088 (51.62%)
[30, Max]	2341 (01.03%)	1 (00.02%)

**Table 4:** PDSCH physical values before and after treatment.

Range (Mbps)	PDSCH (Kbps)	
	Before Treatment	After Treatment
[0, 4]	719 (12.07%)	23,570 (12.42%)
[4, 5]	2053 (34.47%)	4293 (02.26%)
[5, 10]	2053 (34.47%)	26,099 (13.76%)
[10, 20]	1966 (33.01%)	65,427 (34.49%)
[20, 30]	804 (13.50%)	60,242 (31.76%)
[30, 40]	405 (06.80%)	60,242 (31.76%)
[40, 50]	405 (06.80%)	8219 (04.33%)
[50, 60]	9 (00.15%)	8219 (04.33%)
[60, 70]	9 (00.15%)	1359 (00.72%)
[70, Max]	0 (0%)	498 (00.26%)

**Table 5:** PUSCH physical values before and after treatment.

Range (Mbps)	PUSCH (Kbps)	
	Before Treatment	After Treatment
[0, 0.512]	140 (2.28%)	1594 (4.46%)
[0.512, 2]	407 (6.64%)	2747 (7.69%)
[2, 3]	365 (5.96%)	1344 (3.76%)
[3, 10]	2614 (42.66%)	10,460 (29.28%)
[10, 15]	1025 (16.73%)	5384 (15.07%)
[15, 25]	1573 (25.67%)	13,727 (38.43%)
[25, Max]	3 (0.05%)	466 (1.3%)

## Data Availability

The data presented in this study are available on request from the corresponding author.
